# Toxic effect of NiCl_2_ on development of the bursa of Fabricius in broiler chickens

**DOI:** 10.18632/oncotarget.6591

**Published:** 2015-12-13

**Authors:** Shuang Yin, Hengmin Cui, Xi Peng, Jing Fang, Zhicai Zuo, Junliang Deng, Xun Wang, Bangyuan Wu, Hongrui Guo

**Affiliations:** ^1^ Key Laboratory of Animal Diseases and Environmental Hazards of Sichuan Province, Ya'an, Sichuan, China; ^2^ College of Veterinary Medicine, Sichuan Agricultural University, Ya'an, Sichuan, China

**Keywords:** NiCl_2_, lesion, relative weight, cell cycle, apoptosis, Immunology and Microbiology Section, Immune response, Immunity

## Abstract

This study was conducted with objective of evaluating the toxic effects of nickel chloride (NiCl_2_) on development of bursa of Fabricius in broilers fed on diets supplemented with 0, 300, 600 and 900 mg/kg of NiCl_2_ for 42 days by using the methods of experimental pathology, flow cytometry (FCM), and quantitative real-time polymerase chain reaction (qRT-PCR). The results showed that dietary NiCl_2_ in 300 mg/kg and over induced toxic suppression in the bursal development, which was characterized by decreasing lymphocytes histopathologically and relative weight, increasing G_0_/G_1_ phase (a prolonged nondividing state), reducing S phase (DNA replication) and proliferating index, and increasing percentages of apoptotic cells. Concurrently, the mRNA expression levels of bax, cytochrome c (cyt c), apoptotic peptidase activating factor 1 (Apaf-1), caspase-3, caspase-6, caspase-7 and caspase-9 were increased and the bcl-2 mRNA expression levels were decreased. The toxic suppression of bursal development finally impaired humoral immunity duo to the reduction of B lymphocyte population and B lymphocyte activity in the broiler chicken. This study provides new evidences for further studying the effect mechanism of Ni and Ni compoundson B-cell or bursa of Fabricius.

## INTRODUCTION

Nickel (Ni), an essential element of more than one hundred compounds used widely in industry and commerce, is also considered to be a nutritionally essential trace metal for several animal species, microorganisms and plants [1-4]. However, there have been more reports on Ni or Ni compound toxicity than on Ni nutritional deficiency at present. Ni or Ni compounds have been proved to be potentially hazardous to living organisms due to their genotoxicity, immunotoxicity, mutagenicity and carcinogenicity [5-7]. In rats, nickel chloride (NiCl_2_) at a level of 1200 mg/kg can cause weight loss and reduction of food intake [8]. Ling and Leach [9] have reported that diets supplemented with Ni 300 mg/kg or over are toxic to male chicks. It has been also proved that long-term exposure to Ni is deleterious to the upper respiratory tract, skin, kidney, immune system [10-13], embryos, and the breeding system [14-16], and that Ni causes DNA damage and inhibits DNA repair in mammalian cells [17-19]. Ni (II) can directly generate ROS, activate caspase-3 expression, increase caspase-3-like protease activity, and then cause cell death in mice [20, 21]. Our previous studies have shown that dietary NiCl_2_ in excess of 300 mg/kg causes oxidative damage, lesions, immunotoxicity, cytotoxicity, genotoxicity, cytokine content reduction, apoptosis and inflammatory response in the cecal tonsil, spleen, thymus, kidney and intestine [22-37]. In humans, persons are exposed to Ni or Ni compounds *via* food, water, and air produced from sources such as mining, extraction, refining, electroplating, food processing, and waste disposal [38]. After accidently drinking water contaminated with Ni sulfate (NiSO_2_) and NiCl_2_ (1.63g Ni/L), workers can develop acute gastrointestinal and neurological symptoms [39]. Based on the above-mentioned reports, Ni and Ni compound toxic effects on humans and animals, or/and Ni and Ni compound contamination of food and water have been a big problem in the environmental safety and public health.

The bursa of Fabricius has been found only in the bird and is located between the cloaca and the sacrum. It is the primary lymphoid organ, and is necessary for B-cell differentiation, development and maturation, and is responsible for the establishment and maintenance of the B-cell compartment in avian species [40-42]. Thus, bursae may be used as a good model for studies on effects of many factors on B-cell function. However, there have been no reports focused on the toxic effects of NiCl_2_ on bursa of Fabricius in avian species at present.

Therefore, this study was conducted to evaluate toxic effects of NiCl_2_ on bursal development, including histopathological lesions and changes of relative weight, cell cycle phase, percentages of apoptotic bursa cells and the mRNA expression levels of mitochondrial apoptotic pathway-related factors [bcl-2, bax, cytochrome c (cyt c), apoptotic peptidase activating factor 1 (Apaf-1), caspase-3, caspase-6, caspase-7 and caspase-9] by using the methods of experimental pathology, flow cytometry (FCM), and quantitative real-time polymerase chain reaction (qRT-PCR).

## RESULTS

### Clinical observation

From 14 to 42 days of age during the experiment, the feed intake of broilers in the 300 mg/kg, 600 mg/kg, and 900 mg/kg groups began to decline when compared with those in the control group, except the 300 mg/kg group at 14 days of age. From 21 to 42 days of age during the experiment, broilers in the 300 mg/kg, 600 mg/kg, and 900 mg/kg groups showed poor appetite, poor growth and depression. A few broilers showed polypnea. No death was found during the experiment.

### Histopathological observation in the bursa of Fabricius

**Figure 1:**
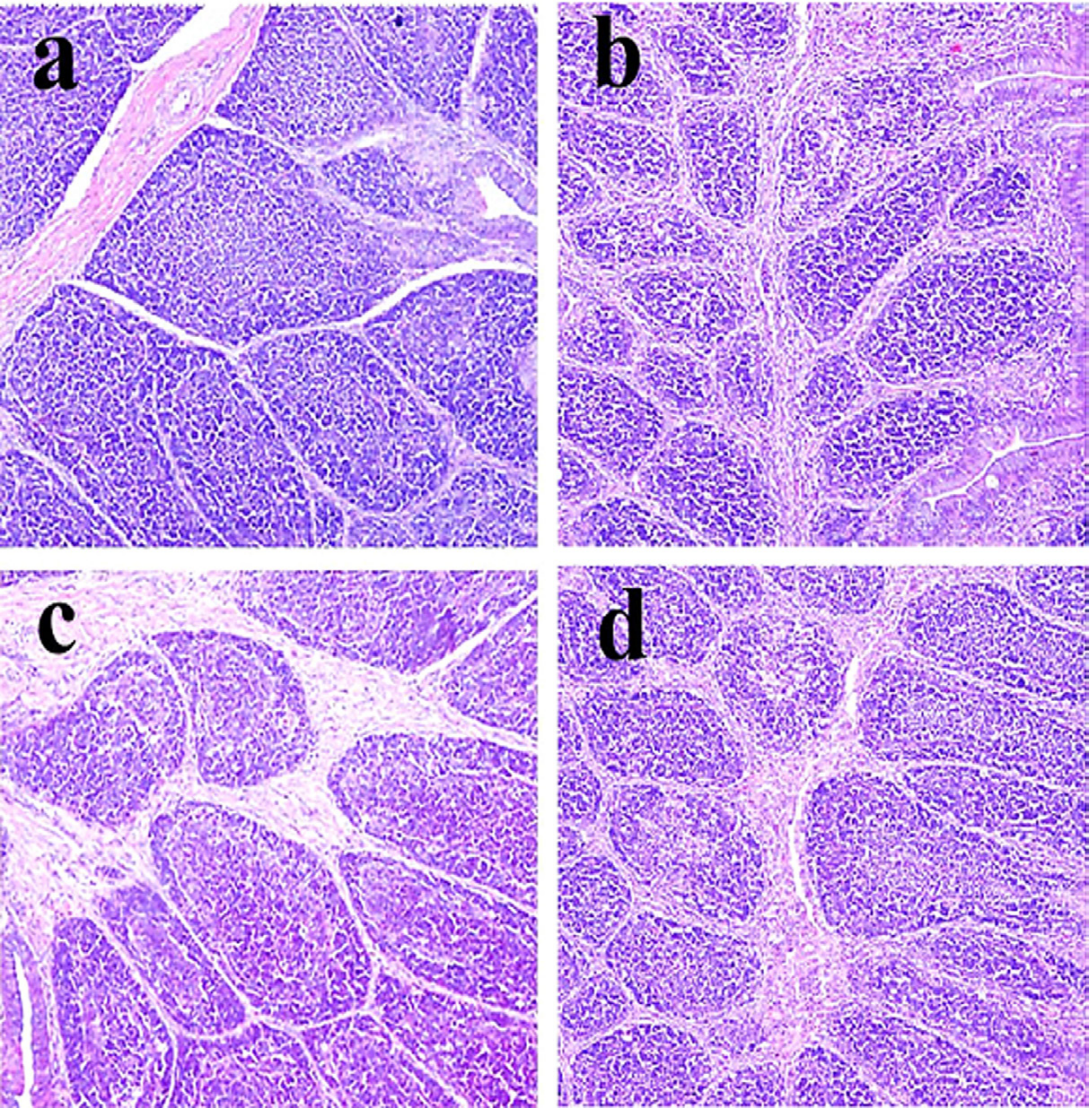
Histopathological changes in the bursa of Fabricius at 14 days of age **a.** Control group. Lymphocytes are slightly reduced in lymphoid follicles of the 600 **c.** and 900 **d**. mg/kg groups when compared with those in the control group. H•E ×200.

**Figure 2:**
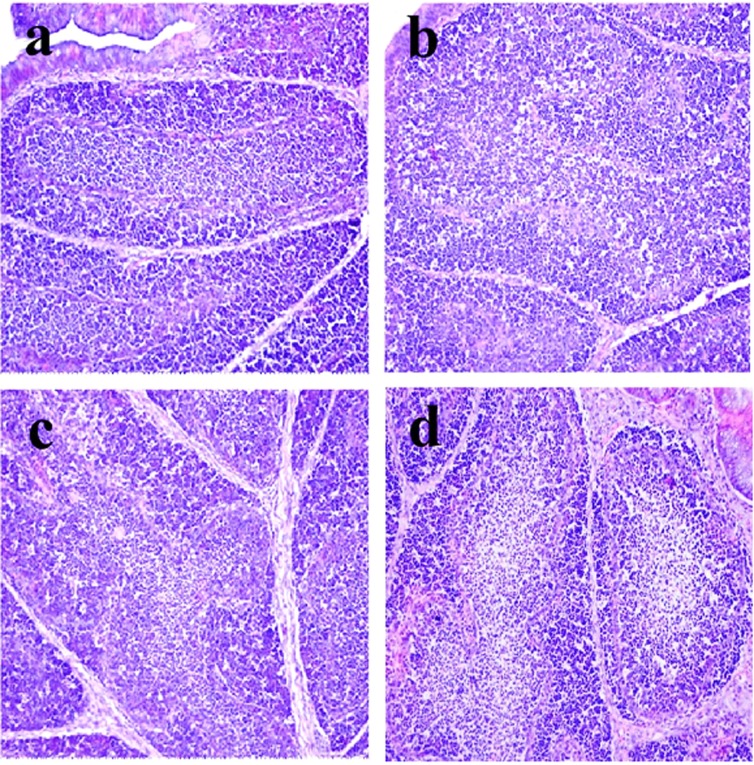
Histopathological changes in the bursa of Fabricius at 28 days of age **a.** Control group. Lymphocytes are reduced in lymphoid follicles of the 300mg/kg group **b**. and are obviously decreased in lymphoid follicles of the 600 **c**. and 900 **d**. mg/kg groups when compared with those in the control group. H•E ×200.

**Figure 3:**
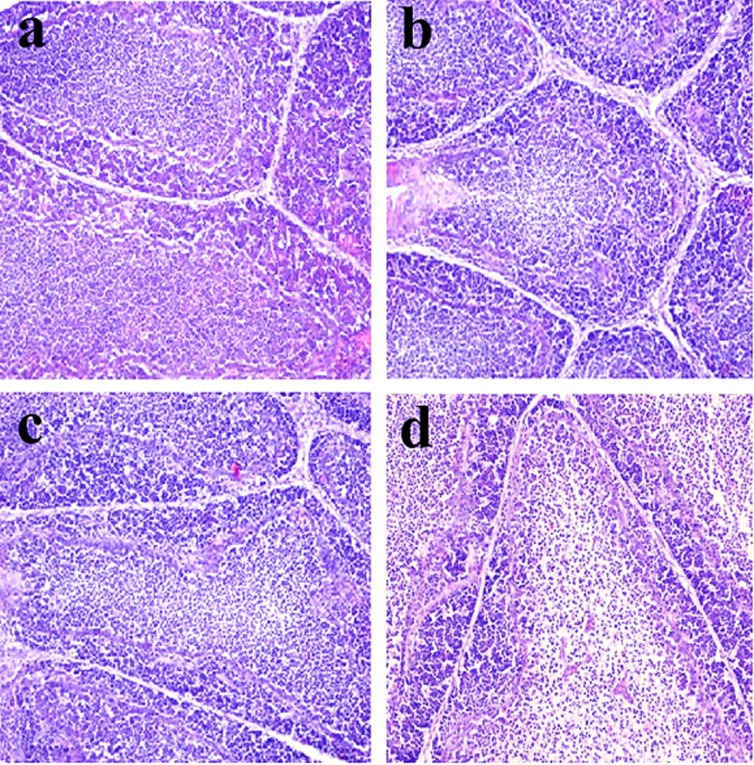
Histopathological changes in the bursa of Fabricius at 42 days of age **a.** Control group. Lymphocytes are obviously reduced in lymphoid follicles of the 300mg/kg group **b**. and are significantly decreased in lymphoid follicles of the 600 **c**. and 900 **d**. mg/kg groups when compared with those in the control group. H•E ×200.

**Figure 4:**
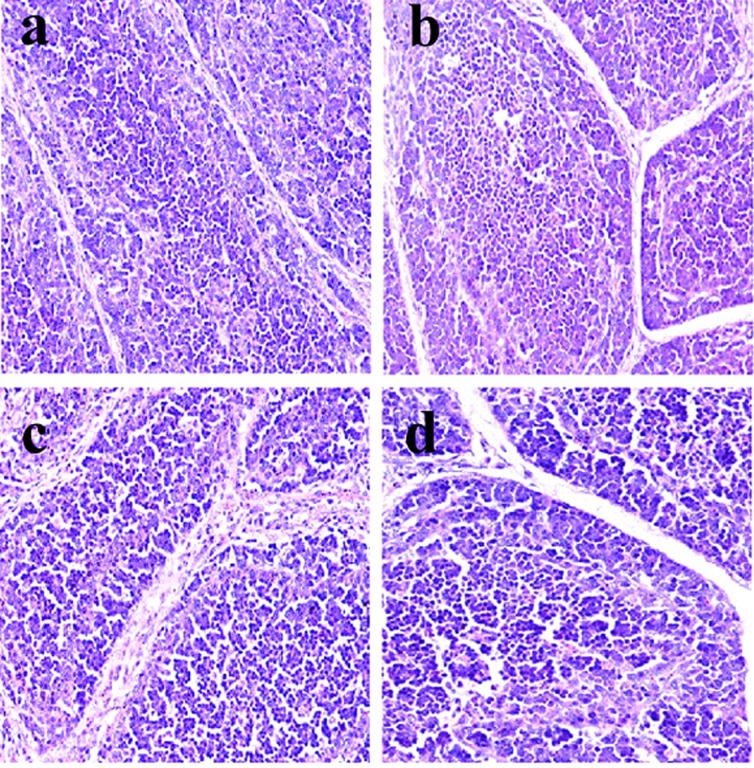
Histopathological changes in the bursa of Fabricius at 14 days of age **a.** Control group. Lymphocytes are slightly reduced in lymphoid follicles of the 600 **c**. and 900 **d**. mg/kg groups when compared with those in the control group. H•E ×400.

**Figure 5:**
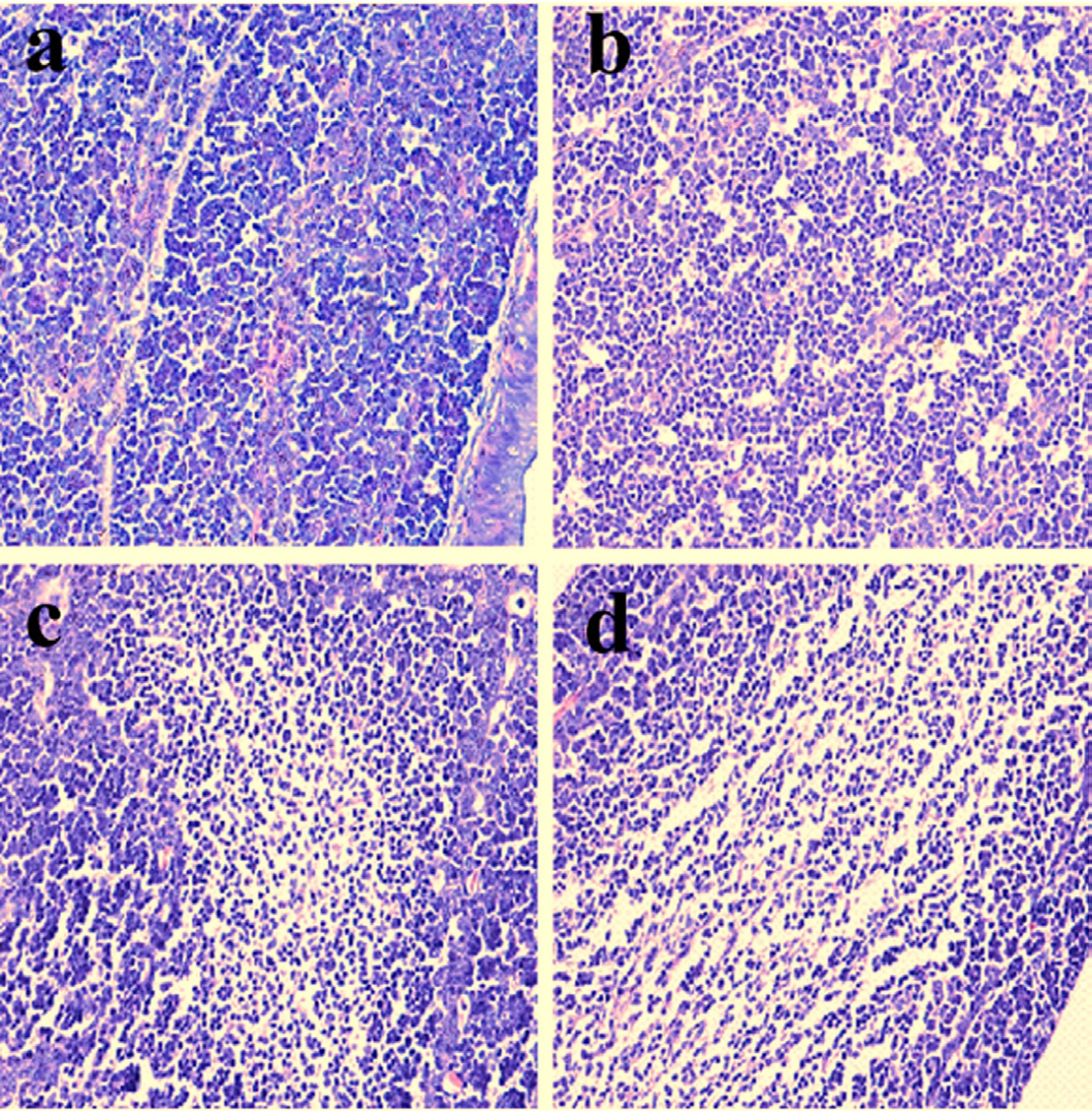
Histopathological changes in the bursa of Fabricius at 28 days of age **a**. Control group. Lymphocytes are reduced in lymphoid follicles of the 300mg/kg group **b**. and are obviously decreased in lymphoid follicles of the 600 **c**. and 900 **d**. mg/kg groups when compared with those in the control group. H•E ×400.

**Figure 6:**
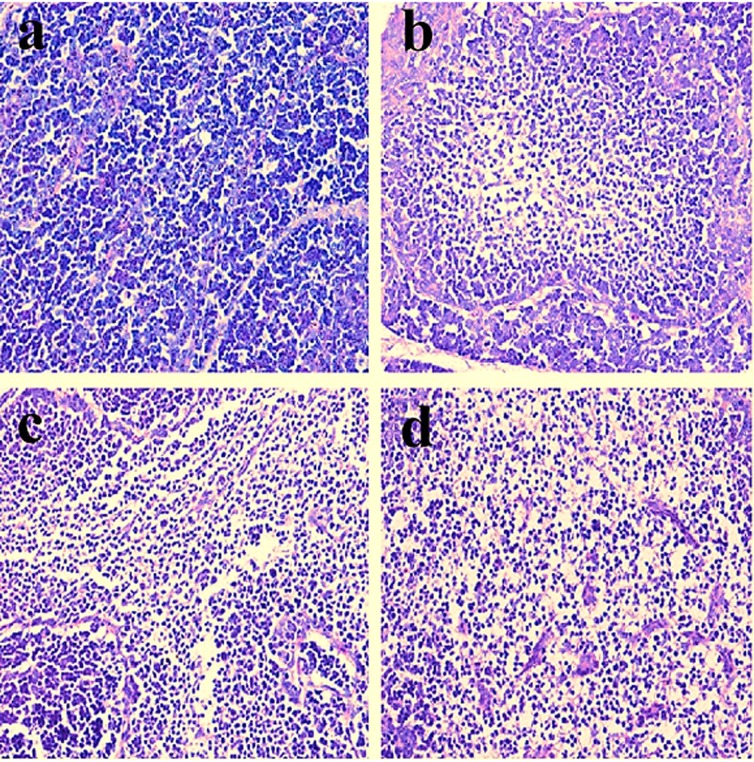
Histopathological changes in the bursa of Fabricius at 42 days of age **a**. Control group. Lymphocytes are obviously reduced in lymphoid follicles of the 300mg/kg group **b**. and are significantly decreased in lymphoid follicles of the 600 **c**. and 900 **d**. mg/kg groups when compared with those in the control group. H•E ×400.

Histopathological changes were observed in the three NiCl_2_-treated groups when compared with those in the control group from 14 to 42 days of age. Also, histopathological changes showed obvious time- and dose-characterization. At 14 days of age, lymphocytes were slightly reduced in lymphoid follicles. At 21 and 28 days of age, lymphocytes were obviously decreased in lymphoid follicles with thinner cortices and wider medullae. At 35 and 42 days of age, lymphocytes were significantly decreased in lymphoid follicles with thinner cortices and wider medullae. The results were shown in Figures 1, 2, 3, 4, 5, 6.

### Changes of relative weight in the bursa of Fabricius

**Figure 7:**
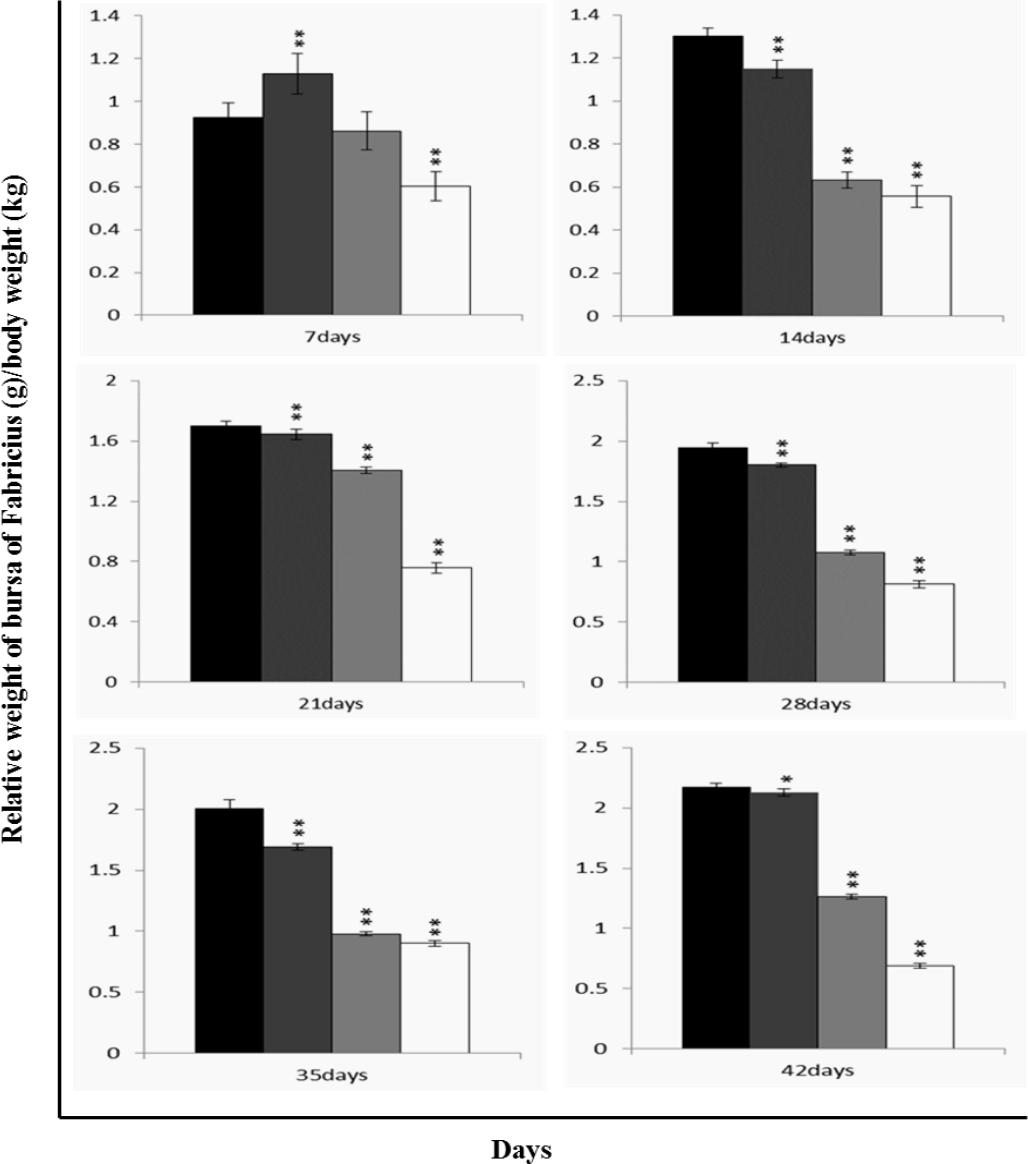
Changes of relative weight [bursa (g)/body weight (kg)] in the bursa of Fabricius Data are the means ± standard deviation (n=5) *p < 0.05, compared with the control group **p < 0.01, compared with the control group.

The relative weight of the bursa was significantly lower (*P* < 0.05 or *P* < 0.01) in the 300 mg/kg, 600 mg/kg and 900 mg/kg groups than those in the control group from 7 to 42 days of age, as shown in Figure 7.

### Changes of cell cycle in the bursa of Fabricius

From 14 to 42 days of age during the experiment, the percentages of G_0_/G_1_ phase (a prolonged nondividing state) were significantly increased (*P* < 0.05 or *P* < 0.01) in the 300 mg/kg, 600 mg/kg and 900 mg/kg groups.

The percentages of the G_2_+M phase were lower (*P* < 0.05 or *P* < 0.01) in the 300 mg/kg, 600 mg/kg and 900 mg/kg groups than those in the control group from 14 to 42 days of age.

The percentages of S phase (DNA replication) were significantly decreased (*P* < 0.01) in the 300 mg/kg, 600 mg/kg and 900 mg/kg groups when compared with those in the control group at 14, 28 and 42 days of age.

The proliferating index (PI) value was markedly lower (*P* < 0.05 or *P* < 0.01) in the 300 mg/kg, 600 mg/kg and 900 mg/kg groups than that in the control group from 14 to 42 days of age.

**Figure 8:**
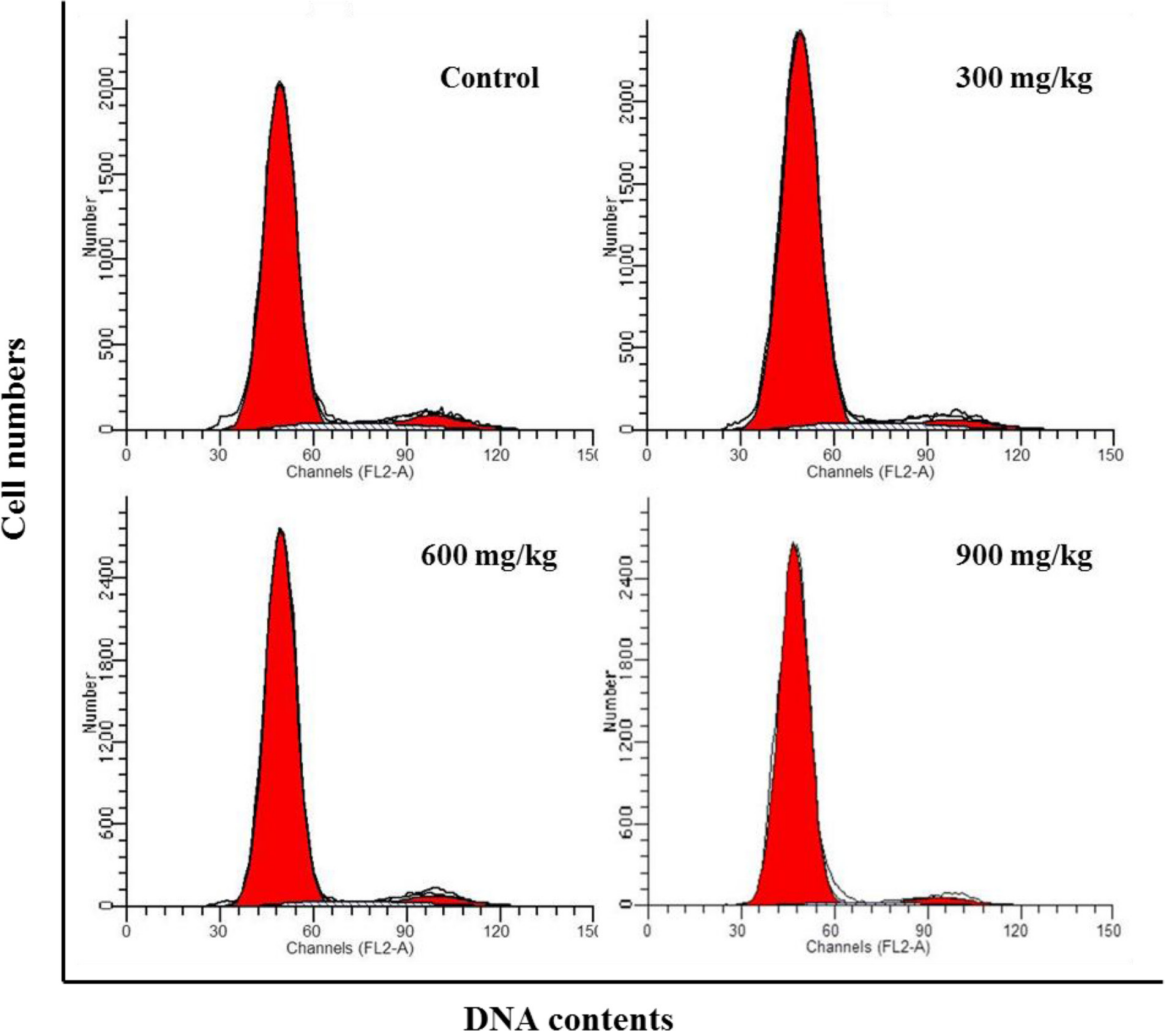
Changes of cell cycle in the bursa of Fabricius by FCM at 42 days of age

**Figure 9:**
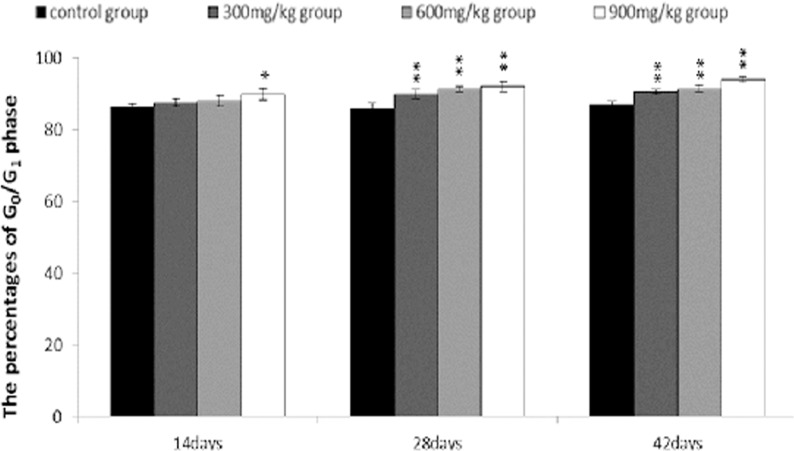
Changes of the percentages of G0/G1 phase in the bursa of Fabricius Data are the means ± standard deviation (n=5) *p < 0.05, compared with the control group **p < 0.01, compared with the control group.

**Figure 10:**
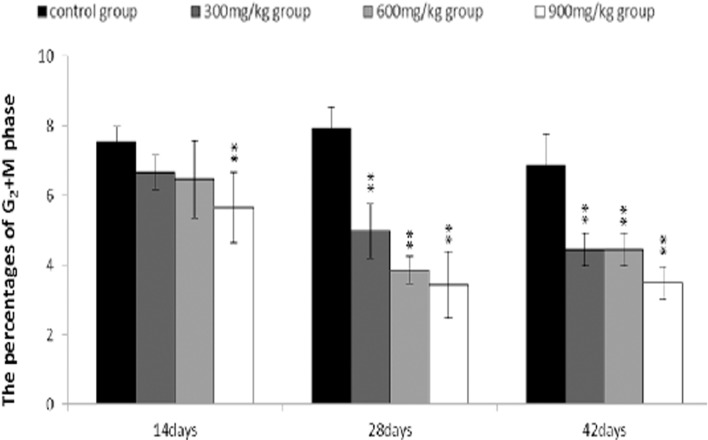
Changes of the percentages of G2+M phase in the bursa of Fabricius Data are the means ± standard deviation (n=5) *p < 0.05, compared with the control group **p < 0.01, compared with the control group.

**Figure 11:**
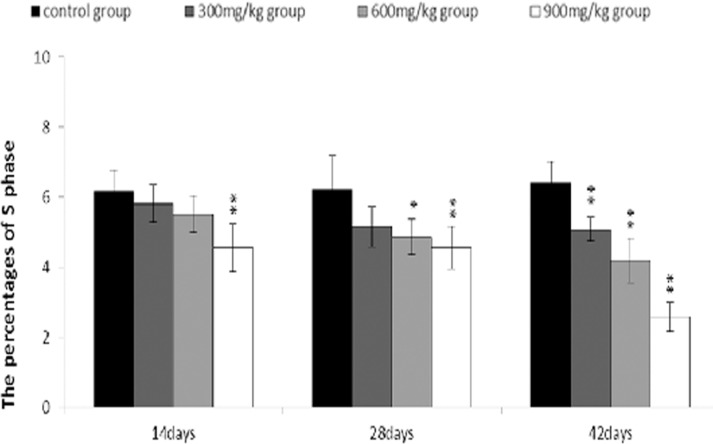
Changes of the percentages of S phase in the bursa of Fabricius Data are the means ± standard deviation (n=5) *p < 0.05, compared with the control group **p < 0.01, compared with the control group.

**Figure 12:**
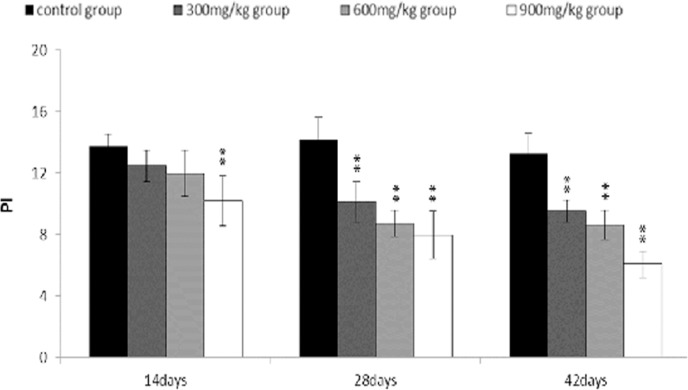
Changes of proliferating index (PI) of bursa of Fabricius PI = [S + (G2 + M)]/[(G0/G1) +S + (G2 + M)] Data are the means ± standard deviation (n=5) *p < 0.05, compared with the control group **p < 0.01, compared with the control group.

The abovementioned results were shown in Figures 8, 9, 10, 11 and 12.

### Changes of apoptosis in the bursa of Fabricius

**Figure 13:**
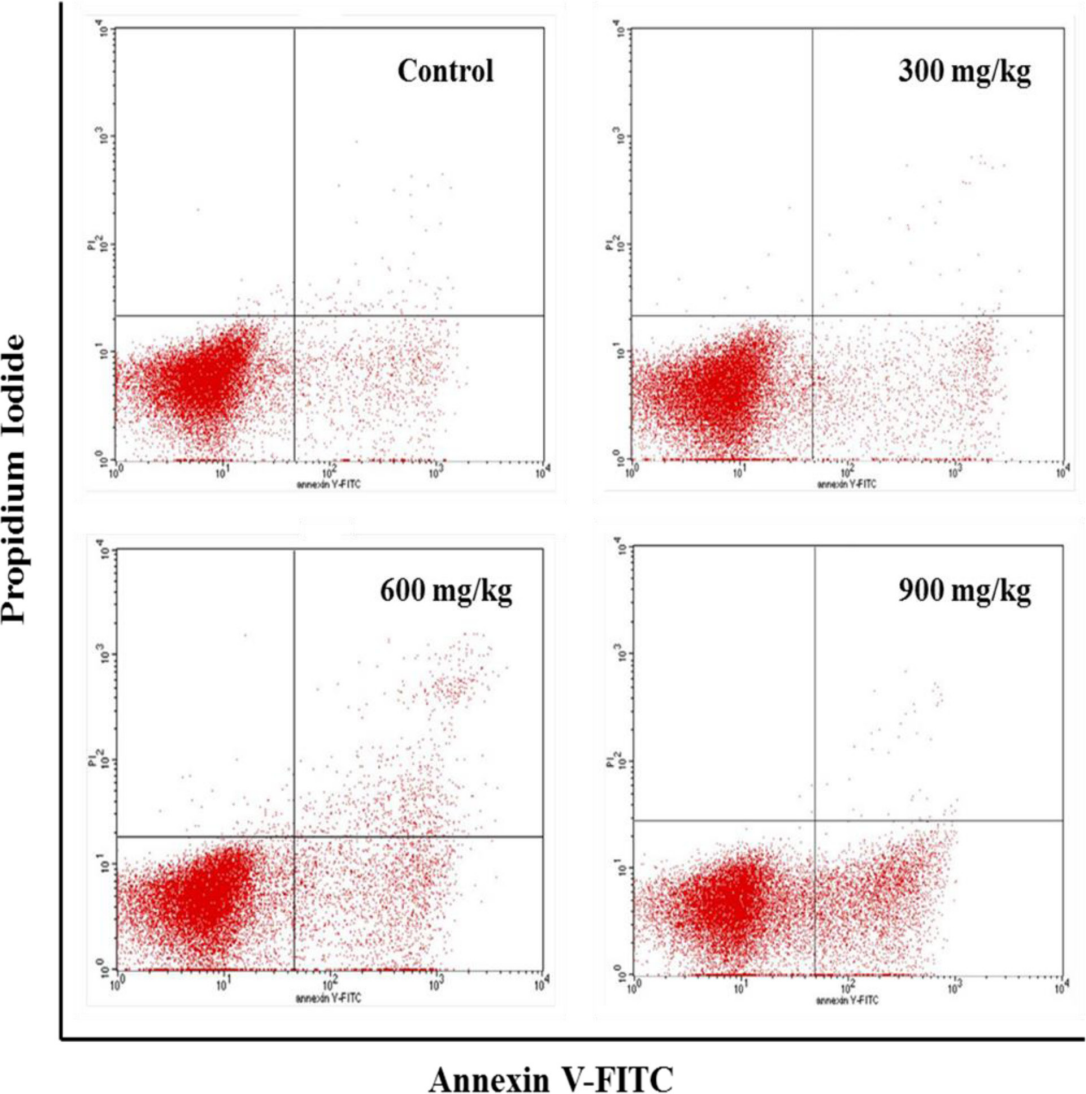
Changes of the apoptosis in the bursa of Fabricius by FCM at 42 days of age

**Figure 14:**
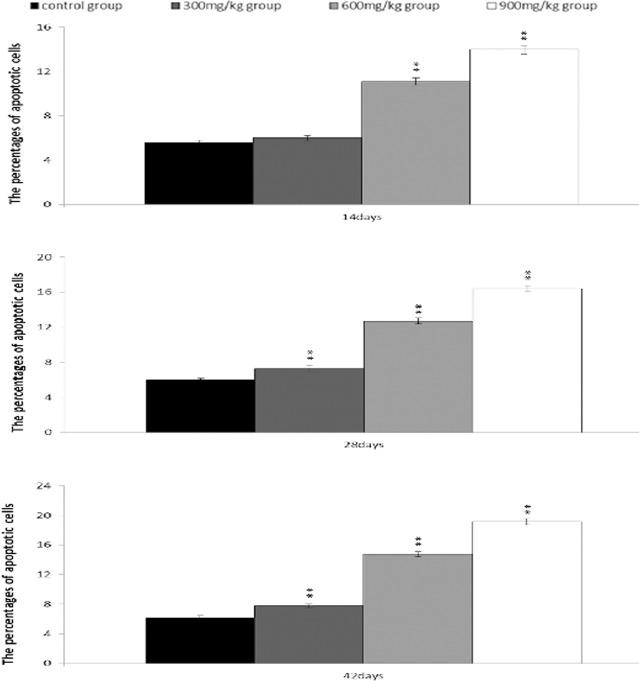
Changes of the percentages of apoptotic cells in the bursa of Fabricius by FCM Data are the means ± standard deviation (n=5) *p < 0.05, compared with the control group **p < 0.01, compared with the control group.

From 14 to 42 days of age during the experiment, the percentages of apoptotic cells in the bursa were increased in the NiCl_2_-treated groups. The apoptotic percentages were significantly higher (*P* < 0.05 or *P* < 0.01) in the 300 mg/kg, 600 mg/kg and 900 mg/kg groups than those in the control group, as shown in Figures 13 and 14.

### Changes of bcl-2, bax, cyt c, Apaf-1, caspase-3, caspase-6, caspase-7 and caspase-9 mRNA expression levels

**Figure 15:**
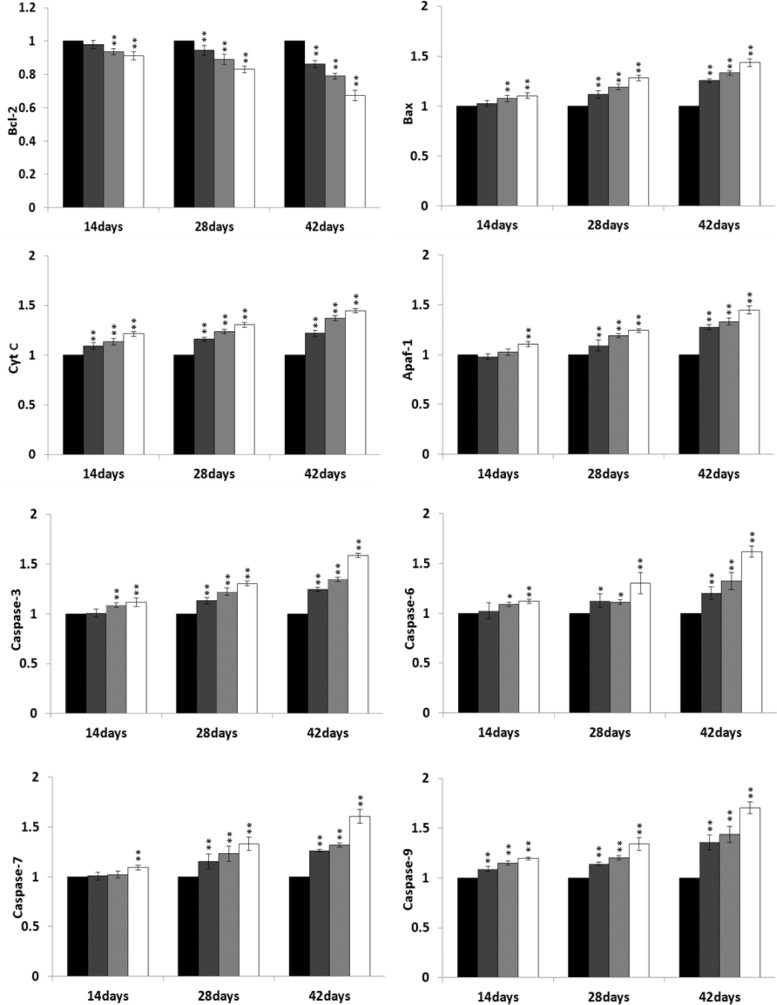
Changes of mRNA expression levels of mitochondrial apoptotic pathway-related factors in the bursa of Fabricius Data are the means ± standard deviation (n=5) *p < 0.05, compared with the control group **p < 0.01, compared with the control group.

At 14, 28 and 42 days of age during the experiment, the bax, cyt c, Apaf-1, caspase-3, caspase-6, caspase-7 and caspase-9 mRNA expression levels were significantly increased (*P* < 0.05 or *P* < 0.01) in the 300 mg/kg, 600 mg/kg and 900 mg/kg groups when compared with those in the control group. However, the bcl-2 mRNA expression levels were significantly lower (*P* < 0.05 or *P* < 0.01) in the NiCl_2_-treated groups than those in the control group from 14 to 42 days of age. The results were shown in Figure 15.

## DISCUSSION

The objective of this study was to evaluate the toxic effects of dietary NiCl_2_ on bursal growth. In the present study, dietary NiCl_2_ in 300 mg/kg and over was indeed found to have toxic effects on bursa of Fabricius, including histopathological lesions (Figures 1, 2, 3, 4, 5, 6), reduced relative weight (Figure 7), arrested cell-cycle (Figures 8, 9, 10, 11 and 12) and increased apoptosis (Figures 13 and 14).

It is well known that relative weight, as a satisfactory measure of nutritive value, can also represent the growth state of organs. In the present study, the relative weight of bursa was used to judge the bursal growth. The relative weight of bursa in NiCl_2_-treated groups was lower than that in control group, implying that dietary NiCl_2_ in 300 mg/kg and over inhibited the bursal development, and then impaired the bursal function, which was consistent with the cell cycle arrest.

In order to reveal how NiCl_2_ induced bursal relative weight reduction or growth suppression, we used FCM to measure the cell cycle of bursal lymphocytes. The results showed that NiCl_2_ induced cell cycle arrest at the G_0_/G_1_ phase, which inhibited damaged cells to stop DNA replication at G_1_ phase, and ultimately resulted in apoptosis of the cells when damaged cells could not be repaired. The above-mentioned observation was supported by the findings that NiCl_2_ decreased bursal lymphocyte numbers in the S phase and reduced proliferating index, and increased percentages of apoptotic lymphocytes of the bursa. It is clear that the cell cycle arrest and apoptosis of bursal lymphocytes are direct evidences of toxic suppression of the bursa of Fabricius induced by dietary NiCl_2_.

Apoptosis is a process of programmed cell death morphologically characterized by chromatin condensation, DNA and nuclear fragmentation, cytoplasmic shrinkage and formation of apoptotic bodies [43]. Inappropriately regulated apoptosis is implicated in an extensive variety of diseases [44]. In our study, the increased apoptotic lymphocytes in the bursa of Fabricius were observed in the three NiCl_2_-treated groups by FCM, which was consistent with the mRNA expression alteration of apoptotic proteins. It was found in the present study that dietary NiCl_2_ increased bax, cyt c, Apaf-1, caspase-3, caspase-6, caspase-7 and caspase-9 mRNA expression levels, and decreased bcl-2 mRNA expression levels, which explained the occurrence of mitochondrial apoptosis pathway in the bursa of Fabricius because increase of pro-apoptotic or reduction of anti-apoptotic genes could push cells down the apoptotic pathway.

The apoptotic process is mediated by the bcl-2 family proteins and performs in Fas pathway or caspase-independent apoptotic pathway which relies on the mitochondrial active control [45]. Bcl-2 family is classified into anti-apoptotic proteins such as bcl-2 and bcl-xL, which reduces the level of cyt c release [46] and pro-apoptotic proteins such as bax and bak, which induces the release of cyt c and a loss of the mitochondrial membrane potential [47]. The cyt c release is the main steps in the mitochondrial apoptosis pathway [48-50]. After being released, cyt c is immediately combined with Apaf -1, and then activates caspase family to induce apoptosis [51]. Caspases are central mediators of apoptosis [52]. Nevertheless, bcl-2-like proteins can prevent bax-induced cell death by blocking cyt c release [53]. The present study showed that mRNA expression levels of bcl-2, bax, cyt c, Apaf1, caspase-3, caspase-6, caspase-7, caspase-9 in the mitochondrial apoptotic pathways had significant difference between control group and NiCl_2_-treated groups. The results of our study are similar to reports that exposure of A549 cells to Ni ferrite nanoparticles [54] results in significant increase in mRNA expression levels of cell cycle checkpoint protein p53 and apoptotic proteins (bax, caspase-3, and caspase-9).

Cell cycle arrest and increased apoptotic percentages contributed to the decrease in bursal lymphocytes, which was supported strongly and directly by histopathological evidences. Histopathologically, bursal lymphocytes were decreased with obvious time- and dose-characterization. Simultaneously, dietary NiCl_2_ has been reported to induce toxicity in the immune organ or tissue, such as spleen, thymus and cecal tonsil [22, 29, 31-33]. B lymphocytes take part in humoral immunity. Thus, the humoral immune function is finally impaired due to the decreased B lymphocyte numbers and reduced B lymphocyte activities in the peripheral blood and lymphatic organ or tissue caused by dietary NiCl_2_.

In conclusion, dietary NiCl_2_ in 300 mg/kg and over induces toxic suppression in the bursal growth by decreasing lymphocytes histopathologically and relative weight, arresting cell cycle, and increasing apoptosis percentage. The toxic suppression of bursal growth finally impairs humoral immunity duo to the reduction of B lymphocyte population and B lymphocyte activity in the chicken. This study provides new evidences for further studying the effect mechanism of Ni and Ni compounds on B-cell or bursa of Fabricius.

## MATERIALS AND METHODS

### Broilers and diets

A total of 280 one-day-old healthy avian broilers were randomly divided into four groups of 70 broilers in each group. The broilers were housed in separate cages with electrically heated units and were provided with water and the control or experimental diets *ad libitum* for 42 days. The commercial broilers' growth cycle is about 42 days, and then they will be put into use for consumption. In this period they grow rapidly and a lot of diet will be consumed, and broilers will easily affected by diet containing metal pollutants (such as Ni). The aim of our study is to evaluate the effect of dietary NiCl_2_ on the broilers in the period of growth.

All experimental procedures involving broilers were approved by Animal Care and Use Committee, Sichuan Agricultural University.

A corn-soybean basal diet formulated by the National Research Council [55] was the control diet. NiCl_2_·6H_2_O (Cheng Du Kelong Chemical Co., Ltd., Chengdu, China) was mixed into the corn-soybean basal diet to produce experimental diets with 300, 600, and 900 mg/kg of NiCl_2_, respectively.

### Clinical signs and the relative weight of bursa of Fabricius

Clinical signs were observed and recorded daily. At 7, 14, 21, 28, 35 and 42 days of age during the experiment, five broilers in each group were humanely killed. Bursa was taken from each broiler and weighed after dissecting connective tissue around the organ. Relative weight of bursa was calculated by the following formula:

Relative weight=organ weight (g)/body weight (kg)

### Histopathological observation

At 7, 14, 21, 28, 35 and 42 days of age, five broilers in each group were humanely killed, and the bursae were fixed in 4% buffered formaldehyde and routinely processed in paraffin. Thin sections (5 μm) of each tissue were sliced from each block and mounted on glass. Slices were stained with hematoxylin and eosin (H.E), and then were examined under an Olympus light microscope.

### Determination of cell cycle in the bursa of Fabricius by FCM

At 14, 28, and 42 days of age, the bursae of five broilers in each group were selected for determination of the cell-cycle by using FCM, as described by Cui et al. [56].

Bursae were immediately removed and ground to form a cell suspension that was filtered through 300-mesh nylon screen. The cells were suspended in 1×binding buffer at a concentration of 1×10^6^ cells/mL after washing the cells twice with cold phosphate buffer solution (PBS) (pH 7.2-7.4). Five hundred microliters of the solution was transferred to a 5mL culture tube and centrifuged (500-1,000 rpm). After removing the supernatant, 5μL 0.25% Triton X-100 and 5μL propidium iodide were added to the tube. Then the cells were gently vortexed and incubated for 30 min at 25°C in the dark. Finally, 500μL PBS was added to each tube, and the contents were analyzed by FCM (BD FACSCalibur) within 45 min.

$$\text{Proliferating index}=\frac{\text{S}+({\text{G}}_{2}+\text{M})}{\left({\text{G}}_{0}/{\text{G}}_{1})+\text{S}+({\text{G}}_{2}+\text{M}\right)}\times 100\%”.$$

### Determination of apoptosis in the bursa of Fabricius by FCM

At 14, 28, and 42 days of age, the bursae of five broilers in each group were selected for determination of the bursal apoptotic lymphocytes using FCM, as described by Peng et al. [57].

The cell suspension was prepared as described in the method of cell of cycle. One hundred microliters of the solution was transferred to a 5 mL culture tube, then subsequently adding 5 μl of Annexin V-FITC and 5 μl of propidium iodide to the tube. The cells were gently vortexed and incubated for 15 min at room temperature (25°C) in the dark. Four hundred microliters of 1×binding buffer was added to each tube and analyzed by FCM within 1 h.

### Detection of apoptotic protein mRNA expression levels by qRT-PCR

At 14, 28, and 42 days of age during the experiment, the bursae from five broilers in each group were respectively stored in liquid nitrogen. Adding liquid nitrogen, the samples were homogenized using a mortar and pestle. Total RNA was extracted from the powder of bursa of Fabricius by RNA isolate (RNAiso) Plus (9108/9109, Takara, Japan). Then the total RNA reverse transcribed into cDNA using a Prim-Script™ RT reagent Kit (RR047A, Takara, Japan) according to the manufacture's introduce. The cDNA was used as a template for quantitative real-time PCR analysis. Sequences for primers of bcl-2, bax, cyt c, Apaf-1, caspase-3, caspase-6, caspase-7 and caspase-9 and β-Actin were obtained from Genbank and NCBI. Primers were designed with Primer 5 software and synthesized at BGI Tech (Shenzhen, China) (Table 1).

**Table 1:** A list of primers in qRT-PCR analysis of mRNA expression of the apoptotic proteins

Gene symbol	Accession number	Primer	Primer sequence(5′-3′)	Product size	Tm (°C)
Bax	XM422067	Forward Reverse	TCCTCATCGCCATGCTCAT CCTTGGTCTGGAAGCAGAAGA	69bp	62
Bcl-2	NM205339	Forward Reverse	GATGACCGAGTACCTGAACC CAGGAGAAATCGAACAAAGGC	114bp	62
Cytc	NM001079478	ForwardReverse	TGTCCAGAAATGTTCCCAGTGC CCTTTGTTCTTATTGGCATCTGTG	138bp	61
Apaf-1	XM416167	ForwardReverse	AAGGGCATAAGGAAGCAATCAA CAGCACAAGAAAGAACAGCACC	156bp	61
Caspase-3	NM204725	ForwardReverse	TGGCCCTCTTGAACTGAAAG TCCACTGTCTGCTTCAATACC	139bp	62
Caspase-6	AF469049	Forward Reverse	TCAGAGGAGACAAGTGCCAGAGT TACTGAATCCTGAACGAGAACTGG	107bp	59
Caspase-7	XM421764	ForwardReverse	CCGAAGTCCTCACTCAGTAACCA TTGCGTGTACCCATTCCTGTT	137bp	59
Caspase-9	AY057940	ForwardReverse	CGAAGGAGCAAGCACGACAG CCGCAGCCCTCATCTAGCAT	130bp	61
β-actin	L08165	ForwardReverse	TGCTGTGTTCCCATCTATCG TTGGTGACAATACCGTGTTCA	178bp	62

For qRT-PCR reactions, 25 μL mixtures were made by using SYBR^®^ Premix Ex Taq^TM^ II (DRR820A, Takara) containing 12.5 μL SYBR^®^ Premix Ex Taq^TM^ II, 1.0μL of forward and 1.0 μL of reverse primer, 8.5 μL ribonuclease (RNase)-free water and 2 μL cDNA. The real-time PCR reaction conditions were consisted of 3 min at 95°C (first segment, one cycle), 10 s at 95°C and 30 s at Tm of a specific primer pair (second segment, 44 cycles) followed by 10 s at 95°C, and 72°C for 10 s (dissociation curve segment) using Thermal Cycler (C1000, BIO RAD, USA). Gene expression values of control group at 14, 28, and 42 days of age were respectively used for gene expression calibration respectively. The results from the qRT-PCR were analyzed with 2^−ΔΔCT^ assay [58] and β-Actin was used as an internal control gene.

### Statistical analysis

The significance of difference among four groups was analyzed by variance analysis, and results presented as mean ± standard deviation (X±SD). The variation was measured by one-way analysis of variance (ANOVA) test of SPSS 16.0 for windows. Statistical significance was considered at *P* < 0.05.
